# Impact of cigarette smoke extract and hyperglycemic conditions on blood–brain barrier endothelial cells

**DOI:** 10.1186/s12987-015-0014-x

**Published:** 2015-07-24

**Authors:** Shikha Prasad, Ravi K Sajja, Jee Hyun Park, Pooja Naik, Mohammad Abul Kaisar, Luca Cucullo

**Affiliations:** Department of Pharmaceutical Sciences, School of Pharmacy, Texas Tech University Health Sciences Center, 1300 S. Coulter Street, Amarillo, TX 79106 USA; Center for Blood Brain Barrier Research, Texas Tech University Health Sciences Center, Amarillo, TX 79106 USA

**Keywords:** Diabetes, Smoking, Alternative, Tight junctions, Glucose transport, P-glycoprotein, Inflammation, Oxidative stress, Hyperglycemia

## Abstract

**Background:**

Diabetes and tobacco smoking are significant public health concerns which have been shown to independently impact the blood–brain barrier (BBB). Since smoking is a risk factor for diabetes and shares some of the common pathological pathways leading to metabolic abnormalities, it is hypothesized that their combination would produce additive or synergistic BBB dysfunction. Therefore, the objective of this study was to assess this hypothesis and evaluate the magnitude of these effects in vitro using hCMEC/D3 cells; a well-established human BBB endothelial cell line.

**Methods:**

Monolayers of hCMEC/D3 cells were exposed to hyperglycemic conditions (HG; 35 mM) or 5% soluble cigarette smoke extracts (CSE, model of mainstream smoke exposure) for 12–24 h. Cells were then harvested for subsequent biochemical analyses. Transendothelial electrical resistance (TEER) and paracellular permeability to florescent dextrans were used to assess monolayer integrity. Analysis of released factors and cytokines was carried out by ELISA. Western blot (WB) analysis/immunofluorescence of relevant molecular targets was carried out. P-gp efflux activity was measured using rhodamine 123.

**Results:**

Immunofluorescence and WB data showed a significant ZO-1 down-regulation by HG and/or CSE over 24 h exposure. CSE in presence of HG produced a synergistic increase in release of vascular endothelial growth factor that was accompanied by decreased TEER and augmented permeability to labeled dextrans in a size-dependent manner. Moreover, CSE increased the expression of GLUT-1 and SGLT-1 in isolated membrane fractions of hCMEC/D3 cells. The effect was amplified by HG. Both, HG and CSE elicited the membrane upregulation of P-glycoprotein (P-gp) expression which however, was not paralleled by a comparable efflux activity. Interestingly, concomitant exposure to HG and CSE evoked a marked upregulation of PECAM-1 and other pro-inflammatory markers including IL-6 and -8, when compared to each condition alone. Moreover, exposure to all tested conditions amplified (to a different degree) cellular oxidative stress response denoted by increased Nrf2 nuclear translocation.

**Conclusion:**

Overall, our results have clearly shown an additive pattern in the release of angiogenic and inflammatory factors following concomitant exposure to HG and CSE. This suggests the involvement of common key modulators in BBB impairment by both CS and HG possibly through the activation of oxidative stress responses.

## Background

Cigarette smoking accounts for 434,000 casualties/year in US and is the leading cause of preventable death. Even though there has been a marginal decline in smoking in recent years, the fact that ≈18% of the US adult population are current smokers is alarming [1]. In 2007 diabetes was the 7th leading cause of death in the US and it is increasing at an alarming rate. One in every three US adults are projected to suffer from diabetes by 2050 [2]. Smoking is a major risk factor for diabetes [3], with 12% of type-2 diabetes mellitus (2DM) cases being attributed to tobacco smoke [4]. Both active and passive smoking not only causes glucose intolerance [5], but significantly increases the risk of diabetes by 45 or 74% in men and women, respectively [6]. Major pathological changes in diabetic patients such as insulin resistance and high levels of glycated hemoglobin (HbA1c) have also been reported in smokers [7]. Both 2DM and smoking have been reported independently to enhance the risk of cerebrovascular and neurological disorders like stroke [1, 8], Alzheimer’s [9, 10], depression [11, 12], cognitive impairment, and vascular dementia [13, 14]; largely due to an increase in reactive oxygen species (ROS) generation [15–17], proinflammatory activity [18, 19], and BBB impairment [20, 21]. However, 2DM and CS—dependent pathophysiological mechanisms underlying these cerebrovascular disorders remain elusive. CS contains over 4,000 chemicals including nicotine and various ROS (e.g., H_2_O_2_, epoxides, nitrogen dioxide, peroxynitrite—ONOO^−^, etc. [22, 23] ) which pass through the lung alveolar wall and raise systemic ROS [24]. At the cerebrovascular level this promotes oxidative damage and BBB breakdown via tight junction (TJ) modification and activation of proinflammatory pathways [25, 26]. Chronic hyperglycemia, a pathogenic alteration characteristic of 2DM, also causes endogenous ROS increase by inhibiting glycolysis and promoting the formation of harmful intermediates such as advanced glycation end products (AGEs) and protein kinase-C pathway (PKC) isoforms, which have DNA and protein damaging effects [27, 28]. At the BBB level, chronic hyperglycemia causes endothelial dysfunction leading to BBB impairment and loss of barrier integrity [28].

Similarly, chronic hyperglycemia has also been reported to alter the expression of a number of BBB functional transporters including facilitative sodium independent glucose transporter-1 (GLUT-1), sodium dependent glucose co-transporter-1 (SGLT-1) and P-glycoprotein (P-gp) [28]. However, the reports are controversial with certain studies reporting a decrease [29, 30] or no change [31–33] in cerebral GLUT-1 and SGLT-1 protein expression, and an increase [34] or unaltered [33] local cerebral glucose utilization. In addition, P-gp expression levels have been reported to decrease [35, 36], increase [37] or remain unaffected [38] in animal diabetic models. Expression and activity changes of P-gp at BBB on smoke exposure have not yet been investigated. Further, despite the epidemiological and translational studies strongly suggesting activation of similar pathophysiological pathways by 2DM and CS, determination and characterization of shared key modulators in BBB impairment lies unexplored. Identification and then targeting of these putative key modulators could help in preventing the initiation of metabolic/cerebrovascular complications in smokers.

Therefore, the objective of our study was to investigate the individual and combinatorial effects of tobacco products and hyperglycemia on BBB endothelium. The experiments were conducted in vitro using a well-characterized human BBB endothelial cell line hCMEC/D3 [39]. Data from this study indicates that hyperglycemia and tobacco smoke (TS) exposure cause dysfunction of BBB endothelium (e.g. impaired tight junction protein expression/distribution, increase in permeability, increase in proinflammatory activity, etc.). Further, our results suggest the existence of similar patterns of endothelial dysfunction in response to TS and hyperglycemia which shows additive or synergistic effects in the majority of our experimental scenarios. Together our data suggest the involvement of shared pathological pathways in TS and hyperglycemia which impact the BBB.

## Methods

### Antibody sources

The antibodies used in this study were obtained from the following sources: rabbit anti-ZO-1 (#D7D12), rabbit anti-ICAM-1 (#4915S) and mouse anti-PECAM-1 (#89C2) from Cell Signaling Technology (Danvers, MA, USA); mouse anti-occludin (#331500) from Life Technologies (Grand Island, NY, USA); mouse anti-glut-1 (#MABS132) and mouse anti-P-gp (517301) from EMD Millipore (Billerica, MA, USA); rabbit anti-SGLT-1 (#ab-14686) from Abcam (Cambridge, MA, USA); rabbit anti-Nrf2 (#sc-722) from Santa Cruz Biotechnology (Santa Cruz, CA, USA); β-actin (#A5441) from Sigma-Aldrich (St. Louis, MO, USA); donkey anti-rabbit (#NA934) and sheep anti-mouse (#NA931) HRP-linked secondary antibodies from GE Healthcare (Piscataway, NJ, USA); goat anti-rabbit (#A11008, A21428) conjugated to Alexa Fluor^®^ 488 and 555, respectively and anti-mouse (#A11001) conjugated to Alexa Fluor^®^ 488 from Invitrogen (Camarillo, CA, USA).

### Reagents

Sterile culture ware was obtained from Fisher Scientific (Pittsburgh, PA, USA), reagents and chemicals were purchased from Sigma-Aldrich or Bio-rad laboratories (Hercules, CA, USA), while Mini-Protean^®^ TGX™ gels 4–15% (#456-1084) from Bio-rad laboratories was used for gel electrophoresis. Dextran-Cascade Blue^®^ (10,000 MW; #D-1976) was obtained from Life Technologies, while Fluorescein isothiocyanate (FITC)-dextran (3,000–5,000 MW; #FD4) and Rhodamine B isothiocyanate (RITC)—dextran (70,000 MW; #R9379) were purchased from Sigma-Aldrich.

### Tobacco smoke preparation

Cigarettes (3R4F) equivalent to full flavor commercial brands with 9.4 mg tar and 0.726 mg nicotine per cigarette were obtained from the University of Kentucky. Cigarette smoke extracts (CSE) was prepared by bubbling eight puffs per cigarette directly into phosphate buffered saline (PBS). This was done in accordance to the ISO/FTC standard smoking protocol (draw of 35 ml, puff duration of 2 s, 1 puff per 60 s), using a single cigarette smoking machine (SCSM, CH Technologies Inc., Westwood, NJ, USA). A 3× concentration (or 300%) of stock solution was first prepared by using three cigarettes. It was then diluted to 5% concentrations in low serum media depending upon the treatment conditions as described later.

### Cell culture

The immortalized hCMEC/D3 endothelial cell line was obtained from Dr. Couraud (INSERM, Paris). These hCMEC/D3 cells (passages no. 28–30) were seeded on collagen-coated cell culture flasks or glass chamber slides (seeding density of 2.5 × 10^4^/cm^2^) and maintained at 37°C with 5% CO_2_ exposure. Cell culture medium consisted of EBM-2 basal medium (Lonza, Walkersville, MD, USA), which was supplemented with 5% FBS (Atlanta Biologicals, Lawrenceville, GA, USA), Fibroblast Growth Factor (Sigma Aldrich), chemically defined lipid concentrate (Life Technologies, Carlsbad, CA, USA), antibiotic/antimycotic (1:1, Atlanta Biologicals, GA, USA) and HEPES (10 mM). The culture medium was changed every other day until the cells reached confluency. Phase contrast microscopy and the expression of characteristic phenotypic markers confirmed the monolayer integrity of the hCMEC/D3 cells at confluency.

### Transwell cell culture setup

Clear polyester transwell inserts (0.4 µm pore size membranes) were seeded with hCMEC/D3 cells (passage no. 28–29) on the luminal side and grown in the culture medium containing EBM-2 basal media and supplements as mentioned above. The wells were coated with collagen prior to seeding. Trans-endothelial electrical resistance (TEER) measurement and phase contrast microscopy was employed to confirm cell layer confluency and integrity.

### Treatment

HCMEC/D3 cells in culture flasks, chamber slides and transwell setup were maintained overnight in media containing 1% FBS with no growth factors (referred to as low serum media). Next day the cell monolayer was exposed to fresh low serum medium of different treatment conditions containing 5.5 mM (normal/control), 35.0 mM d-glucose (HG), normal media containing 5% CSE, HG media containing 5% CSE. These concentrations are based on our previously-published reports.

### Cell viability

Extracellular lactate dehydrogenase (LDH) in the media increases with plasma membrane damage. The LDH levels in the culture medium were measured after 12 and 24 h of experimental exposures to various media conditions—5.5 mM control with or without (w/wo) 5% CSE, 35.0 mM HG w/wo 5% CSE, by a colorimetric enzymatic reaction (Pierce LDH cytotoxicity assay kit, Thermo Scientific, Rockford, IL, USA) in accordance with the manufacturer guidelines.

### Immunofluorescence

HCMEC/D3 cells were seeded in two-well chamber slides and grown as mentioned earlier. Cells were fixed with 16%, methanol free formaldehyde (diluted 1 in 4 in 1X PBS; from Polysciences Inc. # 18814) after specified experimental exposure duration. This was followed by three PBS washes and cell permeabilization using 0.02% Triton 100X. After another three PBS washes, fixed cells were blocked with 5% goat serum in PBS (blocking buffer) at room temperature (RT) for 45 min and incubated overnight at 4°C with primary antibodies prepared in blocking buffer. The following day, cells were incubated for 1 h at RT with Alexa Fluor^®^ 488 or 555 conjugated goat anti-rabbit or anti-mouse antibodies or vice versa, respectively (1:1,000) after three washes with PBS. Thereafter, cells were rinsed, dried and mounted with DAPI in prolonged gold anti-fade mounting media (invitrogen). Mounted slides were examined with EVOS digital inverted fluorescence microscope after overnight drying. Cell slides stained with only secondary antibodies served as negative controls.

### Western blotting

Cells were lysed using a subcellular protein fractionation kit for cultured cells (Thermo scientific, # 78840) as per manufacturer’s guidelines, such that nuclear, cytosolic and membrane fractions were collected. Protein quantification was carried out using Pierce BCA Protein Assay Kit (Thermo Scientific, # 23225). Sample preparation and the entire process was followed as described in our previous report [25]. In brief, denatured samples (15–30 μg) were subjected to SDS-PAGE (4–15% gradient gel) and transferred to PVDF membranes for further blotting. Western blotting was used to measure the protein expression of ZO-1, Occludin, PECAM-1, ICAM-1, GLUT-1, SGLT-1, and P-gp in cell membrane fraction. Nrf-2 protein expression in cytosol vs. nuclear fraction was evaluated. Band densities were analyzed by Image Studio Lite Ver 3.1 and calculated as fold change over control protein expression.

### ELISA

Cell culture media from flasks were collected after 24 h of exposure to treatment conditions (5.5 mM control w/wo 5% CSE, 35.0 mM HG w/wo 5% CSE). These cell culture supernatant samples were then analyzed by Quantikine ELISA kits (R&D systems, Minneapolis, MN, USA) for quantitative determination of vascular endothelial growth factor (VEGF), interleukin-6 (IL-6) and interleukin-8 (IL-8), according to the manufacturer’s protocol.

### BBB integrity

In order to evaluate BBB integrity, our previously-reported method was followed [31]. In brief, a mixture of labeled dextrans in PBS (FITC ~4 kDa, 8 mg/ml; Cascade Blue ~10 kDa, 5 mg/ml; and RITC ~70 kDa, 8 mg/ml) were added to the luminal compartment of the transwells upon treatment exposure of the cells for 24 h. The media was sampled from the abluminal compartment (50 µl) and replaced with the equal volumes of fresh media to maintain appropriate sink conditions. The concentration of each fluorescent dye in the sample was determined by fluorescent measurements at their specific excitation and emission wavelengths. Media samples without dextran and that from abluminal compartments of cell free inserts with dextran added to the luminal compartment, served as references. The permeability measurements were reported as percentage of controls. In addition, we measured TEER (Ω cm^2^) using EVOM 2 (World Precision Instruments, Sarasota, FL, USA), as described earlier [40]. Cell free inserts were also evaluated for TEER for subtraction.

### P-gp efflux activity

To assess the activity of P-gp, the cellular retention of P-gp substrate rhodamine-123 was measured fluorimetrically at excitation/emission wavelengths of 485/535 nm. The cells were cultured as detailed above and were treated for 24 h with 35.0 mM d-glucose (HG), normal media containing 5% CSE, HG media containing 5% CSE, apart from 5.5 mM (normal/control) exposure. At the end of 24 h, cells were washed with cold Hank’s balanced salt solution (HBSS) without calcium and magnesium (Life Technologies) and incubated on ice with rhodamine123 (10 µg/ml) in efflux buffer for 1 h. The efflux buffer consisted of 10 mM HEPES, 1% BSA in EBM-2 basal medium. Cells were subsequently washed with pre-warmed HBSS and incubated in efflux buffer at 37°C for 30 min under gentle agitation. Finally, cells were rinsed with ice-cold PBS and lysed with 0.3% triton X-100. The fluorescence of cell lysates was measured and normalized to protein content of the sample.

### Statistical analysis

Data from all experiments were expressed as mean ± standard deviation (SD) and analyzed by one-way ANOVA using GraphPad Prism 6 Software Inc. (La Jolla, CA, USA). Post hoc multiple comparison tests were performed with Tukey’s test. *P* value <0.05 was considered statistically significant.

## Results

### Exposure to hyperglycemia and/or cigarette smoke extract does not affect hCMEC/D3 cell viability

**Figure 1:**
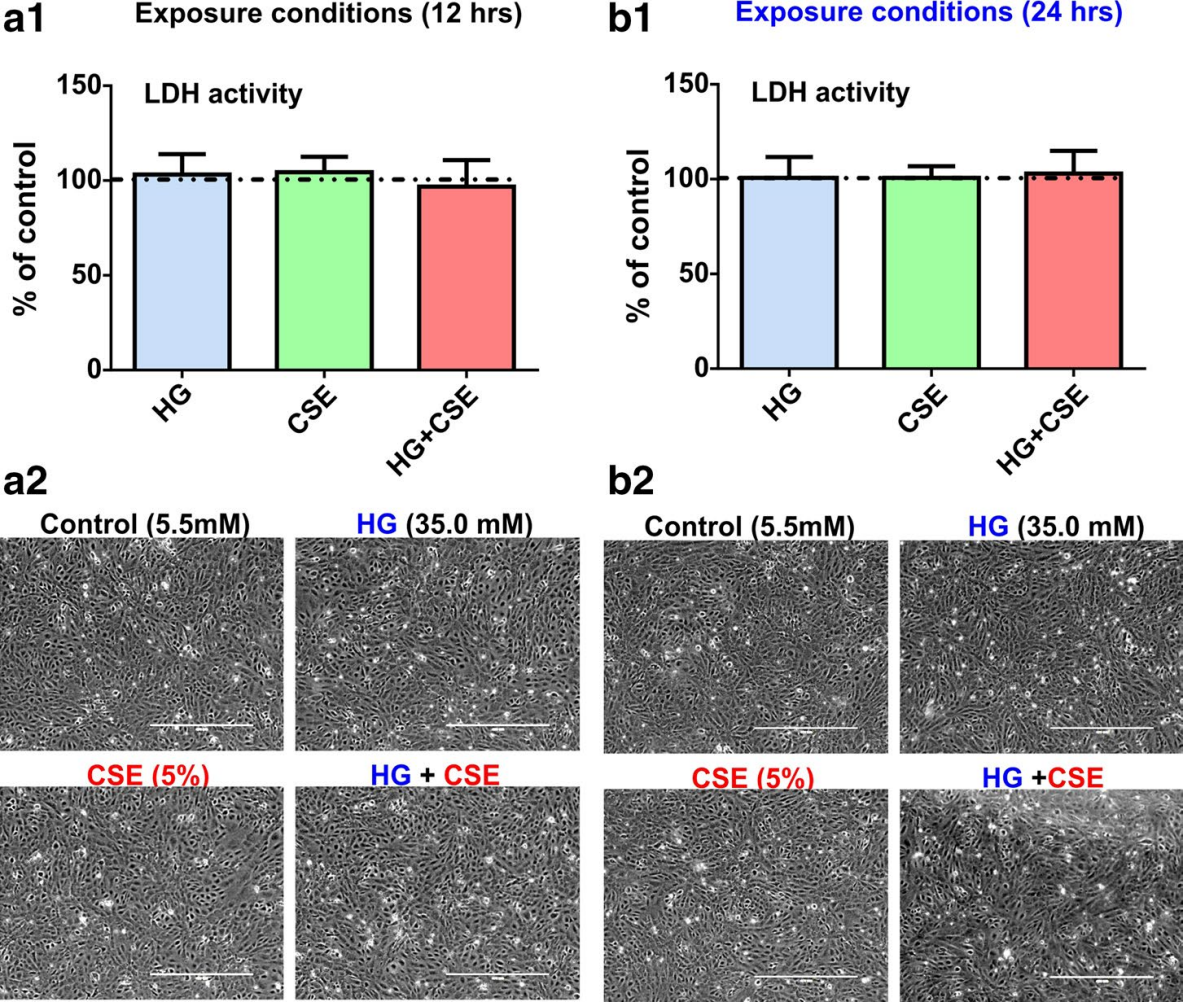
Effects of hyperglycemic conditions and cigarette smoke extract exposure on hCMEC/D3 cell viability. Lactate dehydrogenase (LDH) release, a measure of cytotoxicity from the D3 cell monolayers at 12 h (**a**) and 24 h (**b**) following exposure to HG, CSE and HG + CSE conditions vs. controls. Data are expressed as mean ± SD (% control). Phase contrast microscopic images (10×, *scale* 400 µm) of the hCMEC/D3 monolayers clearly showed a lack of cell toxicity under the experimental conditions tested above. n = 3 biological replicates.

Endothelial cell viability was determined by assessing the release of LDH into the medium in response to specific test culture conditions vs. controls. As shown in Figure 1a1, b1, 12 and 24 h cell exposure to 5% diluted CSE from 3R4F regular cigarettes, 35.0 mM HG media and a co-exposure to hyperglycemia and CSE (HG + CSE) did not cause any significant cellular toxicity as demonstrated by LDH release. Cytotoxicity detection results were further confirmed by morphological inspection. Phase contrast microscopy images (Figure 1a2, b2) showed that hCMEC/D3 cell maintained an intact fully confluent monolayer without any loss of contacts between adjacent cells despite exposure to CSE, HG or HG + CSE. No difference was observed following 12 h or 24 h exposure to the test conditions vs. controls.

### Exposures to hyperglycemia and/or cigarette smoke extract impact ZO-1 protein expression and distribution

**Figure 2:**
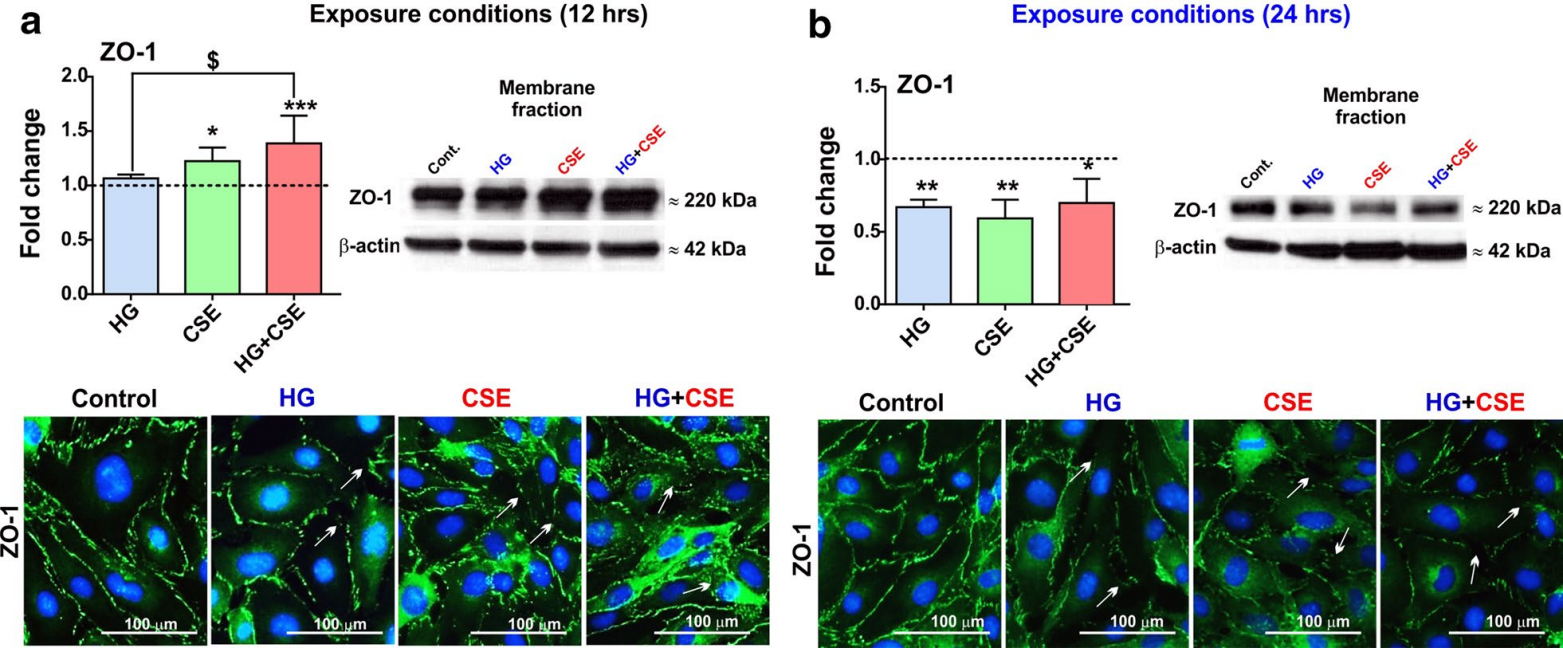
Exposure to hyperglycemic conditions, cigarette smoke extract and both combined impact expression and distribution of tight junction protein ZO-1. **a** 12 h exposure to CSE and HG + CSE increases ZO-1 expression in HCMEC/D3 as clearly shown by Western blot and immunofluorescence analysis of cell monolayers. However, protein distribution in the membrane was significantly altered. **b** By contrast to 12 h, 24 h exposure reversed the previous effect on ZO-1 expression which appeared to be down-regulated. As clearly shown by immunofluorescence analysis (both 12 and 24 h), there was a significant additive disruptive effect on ZO-1 distribution when HG and CSE were combined. n = 3–4 biological replicates. *p < 0.05, **p < 0.01, ***p < 0.001 vs. control; ^$^ *p* < 0.05 vs. HG.

Immunofluorescence analysis of BBB endothelial cell monolayer revealed an initial increase in ZO-1 expression (at 12 h) followed by a significant loss of ZO-1 relative to control (at 24 h) at cell–cell junctions on exposure to HG, CSE and HG + CSE. Despite the increase immunoreactivity to ZO-1 observed following the initial 12 h of exposure to the test conditions, the immunofluorescence images revealed a dysregulation in ZO-1 protein alignment at cell–cell junction. These results were further confirmed by Western blot analysis of the corresponding membrane fractions (Figure 2a, b) in which an increase in ZO-1 membrane expression due to CSE (*p* < 0.05) and HG + CSE (*p* < 0.001) at 12 h was followed by a decrease of ZO-1 membrane expression at 24 h under the same experimental conditions (HG, CSE (*p* < 0.01) and HG + CSE (*p* < 0.05). However, under the same experimental conditions occludin expression levels at 12 and 24 h were largely unchanged when compared to controls (data not shown).

### Effects of hyperglycemia and/or cigarette smoke extract on hCMEC/D3 endothelial monolayer integrity

**Figure 3:**
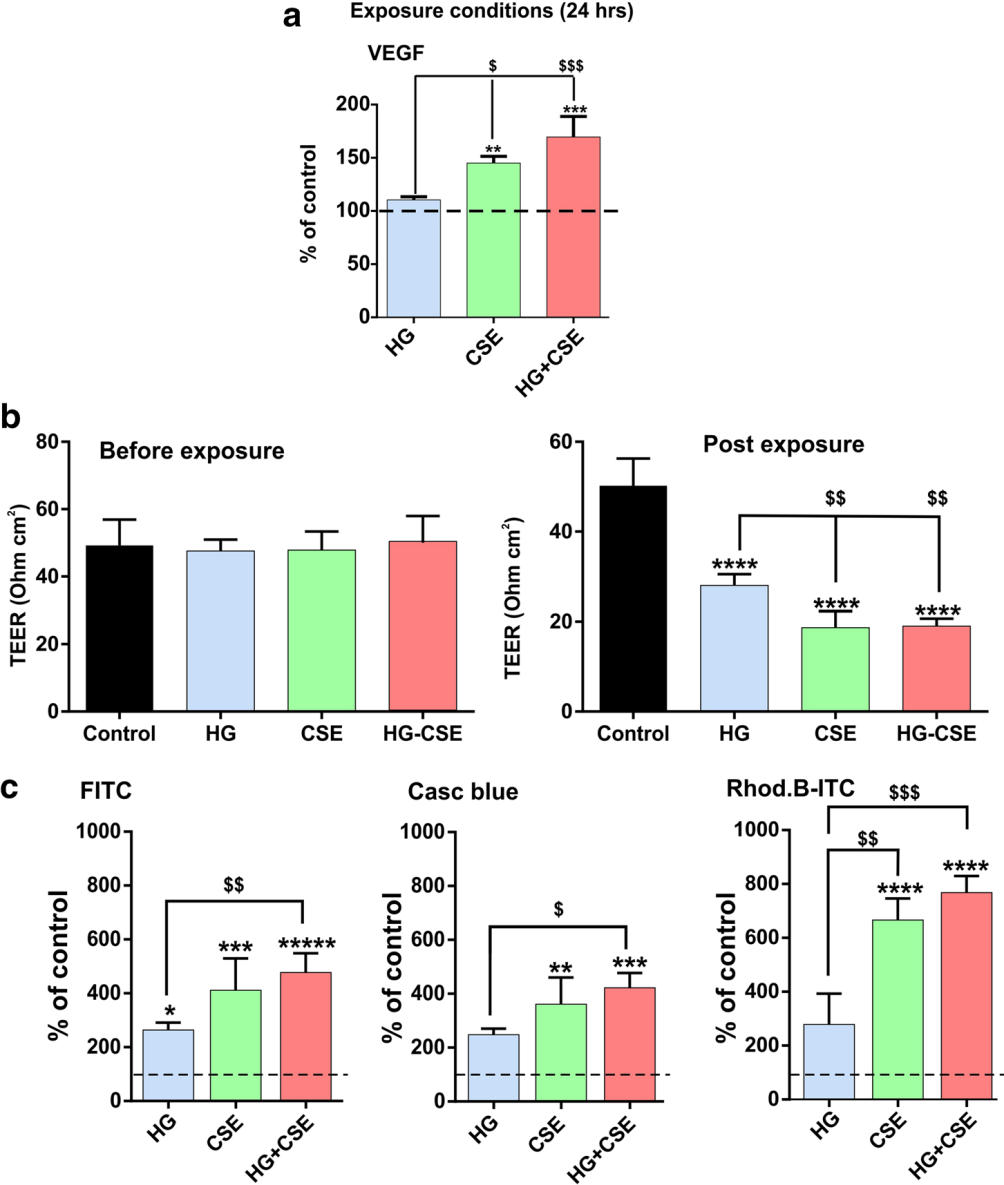
Effects of hyperglycemic conditions, cigarette smoke extract and both combined exposure on BBB endothelial monolayer integrity. **a** CSE and HG-CSE but not HG elicited the release of VEGF (*p* < 0.01 for CSE, *p* < 0.001 for HG-CSE). **b** HG, CSE and HG + CSE induced a significant decrease of trans-endothelial electrical resistance (TEER). This is in agreement with a corresponding increased permeability to dextran molecules Rhod.B-ITC (70 kDa), Cascade blue (10 kDa) and FITC (3–4 kDa) as shown in **c**. n = 5 biological replicates. *p < 0.05, **p < 0.01, ***p < 0.001, ****p < 0.0001, *****p < 0.00001 vs. control and ^$^ *p* < 0.05, ^$$^ *p* < 0.01, ^$$$^ *p* < 0.001 vs. HG.

Vascular endothelial growth factor (VEGF) is known to induce angiogenesis as well as cause permeabilization of blood vessels [41]. As shown in Figure 3a, CSE and HG + CSE triggered a significant endothelial release of VEGF (*p* < 0.01 for CSE, *p* < 0.001 for HG + CSE). In contrast, HG exposure alone did not elicit any statistically significant VEGF release although a modest increase was noted. Our data also clearly showed an additive effect on VEGF release in cell cultures co-exposed to HG and CSE together, with a larger effect than cultures exposed to either HG or CSE alone. As expected, alteration of ZO-1 protein expression/distribution as well as increase in VEGF impacted the integrity of the BBB endothelial monolayer. This is clearly demonstrated by TEER measurement before (average values were 49.25 ± 7.63 SD; 47.6 ± 3.36 SD; 47.83 ± 5.52 SD; 50 ± 7.93 SD Ohm.cm^2^ for control, HG, CSE and HG + CSE, respectively) and after 24 h experimental exposure (50.20 ± 6.05 SD; 28.00 ± 2.53 SD; 18.67 ± 3.67 SD; 19 ± 1.67 SD Ohm.cm^2^ for control, HG, CSE and HG + CSE, respectively); and permeability measurement to dextran molecules across the endothelial monolayer established on Transwell supports. TEER values decreased significantly after 24 h of exposure to HG, CSE and HG + CSE (*p* < 0.0001) in comparison to controls (Figure 3b). Note the increased paracellular flux (luminal to abluminal) of fluorescent dextrans shown in Figure 3c. Increased permeability to 70 kDa dextran (Rhod.B-ITC) and 10 kDa dextran (Casc blue) were noted in transwells exposed to HG, CSE and HG + CSE although results were statistically significant (*p* < 0.01) only for the last two conditions. On the other hand, permeability to 3–4 KDa, lower molecular weight dextran (FITC) was significantly increased when compared to controls in all the exposure conditions mentioned above. Further, loss of barrier functions was considerably more severe in response to HG + CSE exposure and followed the same pattern observed for the release of VEGF.

### Exposure to hyperglycemia and/or cigarette smoke extract affect expression of glucose transporters in the membrane fraction of blood–brain barrier endothelial cells

**Figure 4:**
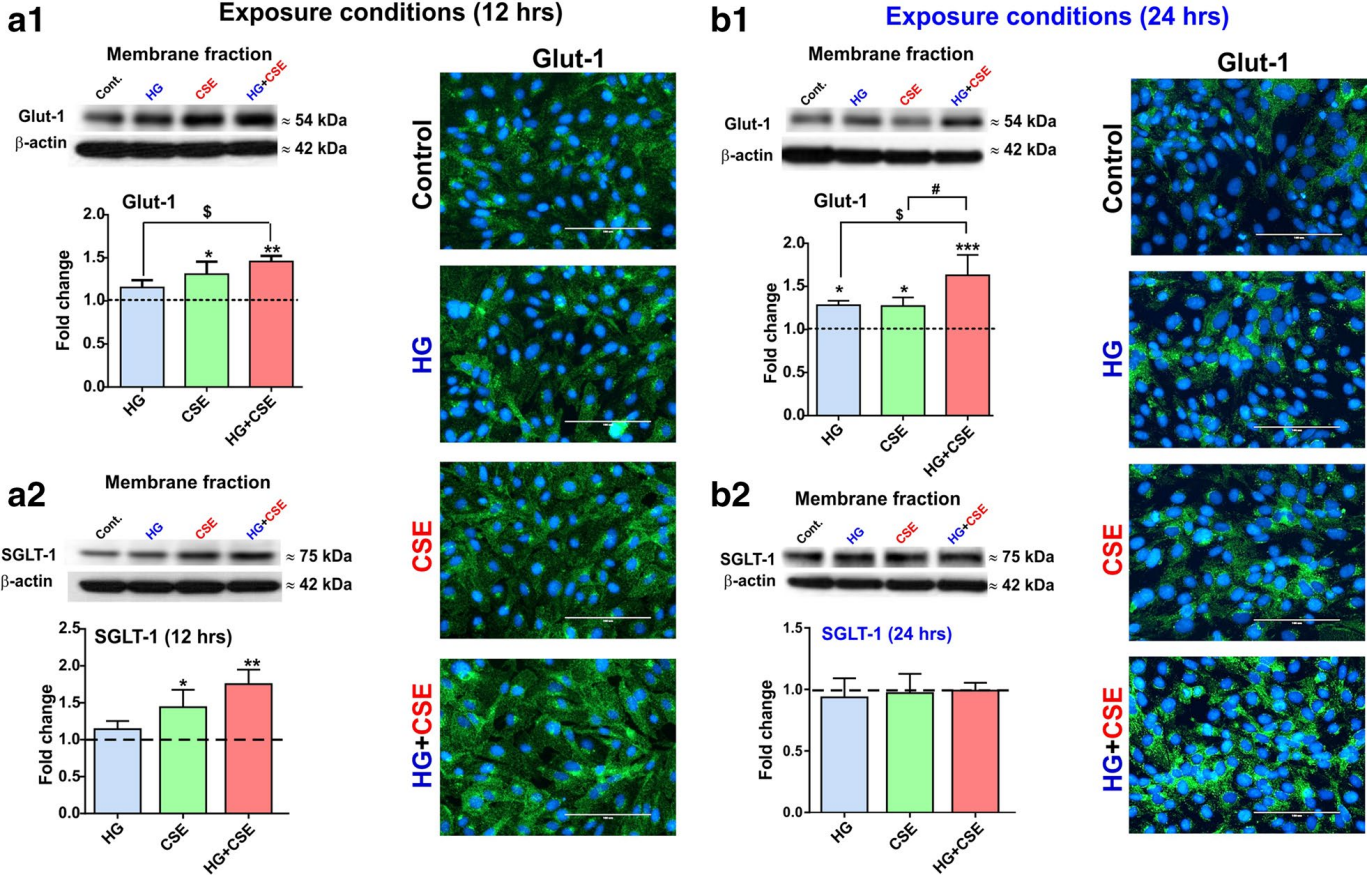
Hyperglycemic conditions, cigarette smoke extract and both combined affect expression of glucose transporters in membrane fractions of BBB endothelial cells. Sodium-independent and dependent glucose transporter proteins- GLUT-1 (**a1**) and SGLT-1 (**a2**) were significantly upregulated by CSE and HG + CSE in an additive pattern. GLUT-1 upregulation persisted at 24 h exposure (**b1**) however, SGLT-1 membrane expression returned to levels comparable to that of controls (**b2**). n = 3–4 biological replicates. *p < 0.05, **p < 0.01, ***p < 0.001 vs. control; ^$^ *p* < 0.05 vs. HG and ^#^ *p* < 0.05 vs. CSE.

Western blot analysis of the endothelium membrane fractions following 12 h exposure to CSE and HG + CSE revealed a significant up-regulation of both sodium independent and dependent glucose transporter proteins—GLUT-1 and SGLT-1, respectively (*p* < 0.05). No significant changes in GLUT-1 and SGLT-1 expression were noted in hCMEC/D3 cultures exposed to HG (Figure 4a1, a2). The effect persisted at 24 h exposure wherein GLUT-1 upregulation was significant even in HG-exposed cultures (*p* < 0.05) (Figure 4b1). By contrast, SGLT-1 upregulation was transient and returned to baseline levels at 24 h exposure in all tested conditions (Figure 4b2). Western blot results were confirmed by immunofluorescence analysis revealing similar uptrends of GLUT-1 expression.

### P-glycoprotein expression and activity in membrane fraction of blood–brain barrier endothelial cells is upregulated by co-exposure to hyperglycemia and cigarette smoke extract

**Figure 5:**
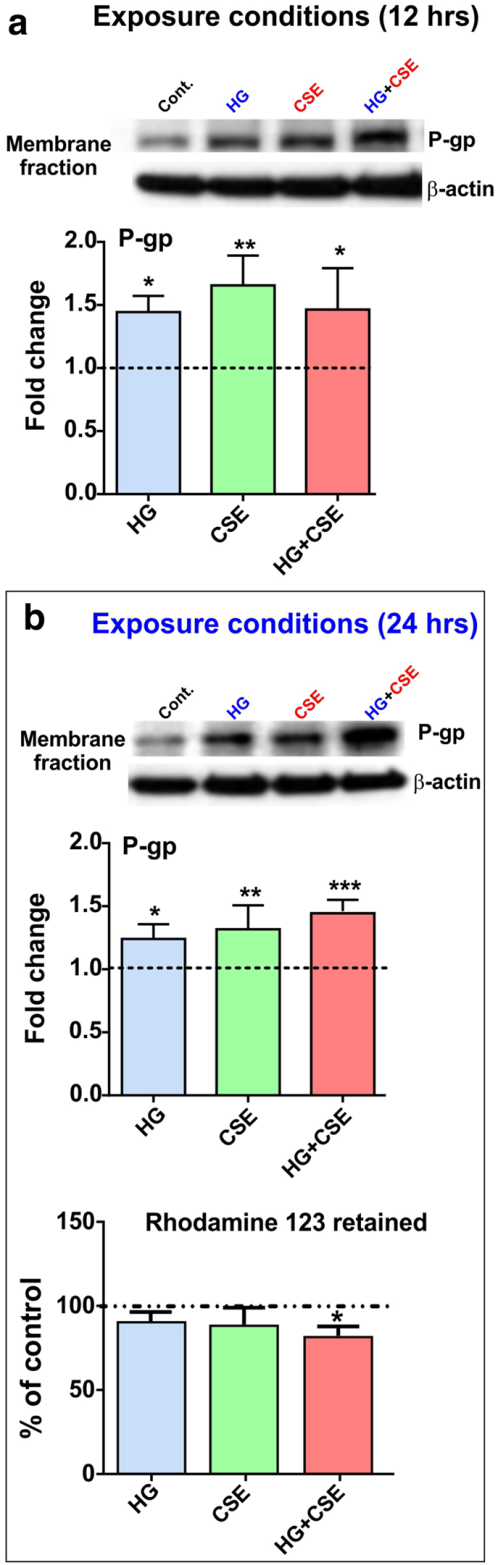
HCMEC/D3 P-gp protein expression and activity is upregulated by exposure to hyperglycemic conditions, cigarette smoke extract and both combined. **a** P-gp expression in the membrane fraction was significantly upregulated following 12 h exposure to HG (*p* < 0.05), CSE (*p* < 0.01) and HG + CSE (*p* < 0.05) when compared to the corresponding controls. **b** Effect on membrane P-gp expression persisted at 24 h exposure under all tested conditions. However, a significant increase in efflux activity was observed only in endothelial cultures exposed to HG + CSE. (*p* < 0.05; n = 4 biological replicates). *p < 0.05, **p < 0.01, ***p < 0.001 vs. control.

As revealed by Western blot analyses of the endothelial membrane fractions from hCMEC/D3 cultures exposed to HG, CSE and HG + CSE, P-gp expression was upregulated at both 12 and 24 h in all the tested conditions (*p* < 0.05; see Figure 5a, b) when compared to the corresponding controls. However, the increase in P-gp expression after 24 h exposure to HG or CSE did not translate into a comparable increase of rhodamine 123 efflux. The effect became statistically significant only when the cell monolayers were co-exposed to both HG and CSE (*p* < 0.05; see Figure 5b).

### Hyperglycemia and/or cigarette smoke extract promote pro-inflammatory activity and anti-oxidant defense response mechanisms in hCMEC/D3 endothelial cells

**Figure 6:**
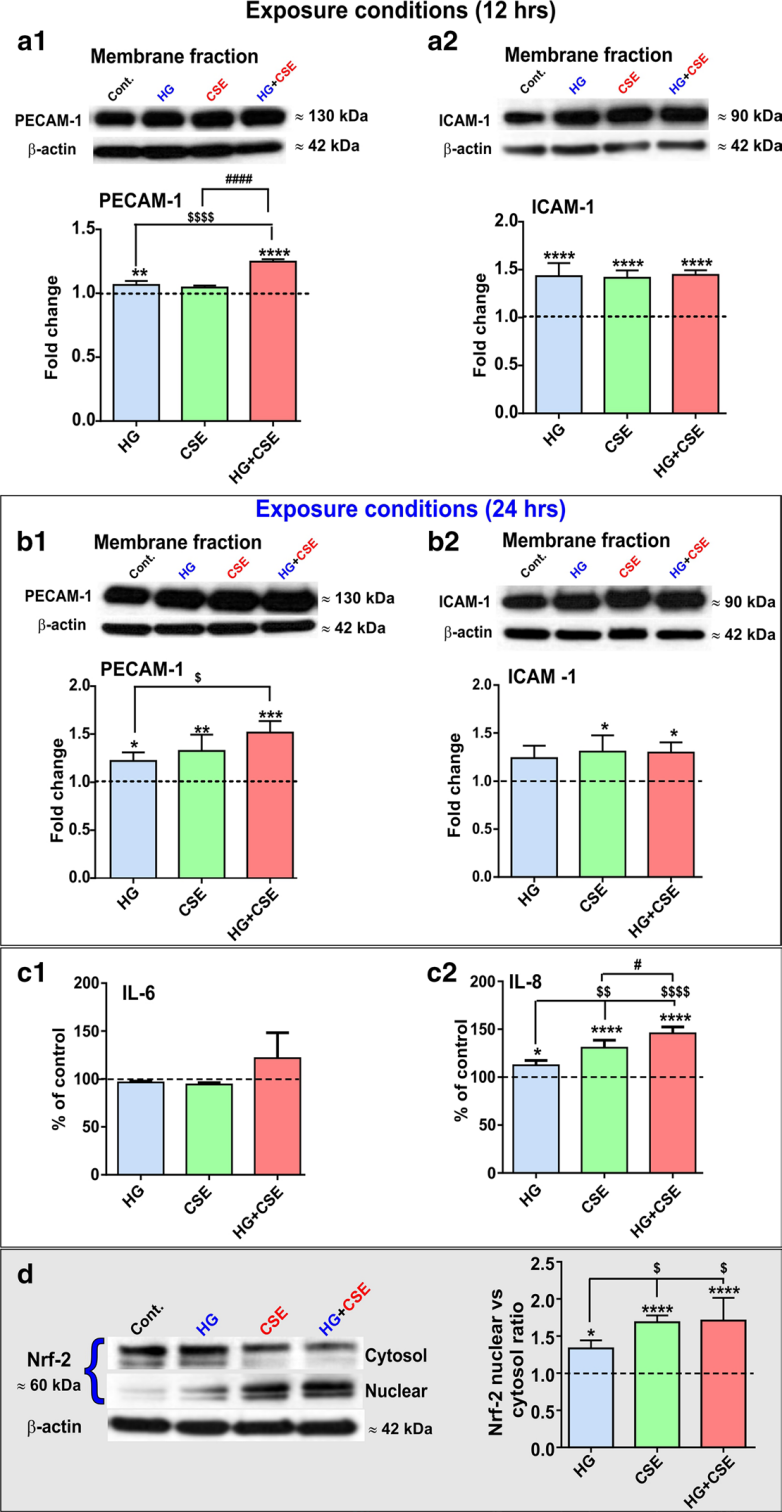
Exposure to hyperglycemic conditions, cigarette smoke extract and both combined promotes the pro-inflammatory and anti-oxidant defense activation in HCMEC/D3 BBB endothelial cells. **a1** PECAM-1 expression in membrane fraction was significantly increased only in endothelial cultures following 12 h exposure to HG and HG + CSE. The magnitude of the effect significantly increased at 24 h exposure showing a consistent additive pattern between tested conditions (**b1**). **a2** 12 h HG, CSE and HG + CSE triggered upregulation of ICAM-1 membrane fraction. By contrast, although statistically significant, the upregulation of ICAM-1 did not show a longitudinal response pattern to the tested conditions (**b2**). **c1**, **c2** Release of IL-6 modestly increased in HG-CSE treated cultures while an additive and more robust increased in IL-8 release was observed under the same conditions at 24 h. **d** Representative Western blot showing the translocation of Nrf-2 from cytosol to nucleus of HCMEC/D3 cells in response to the above mentioned tested conditions. The graph bars clearly show the nuclear vs. cytosol ratios of Nrf-2 under the tested conditions. n = 3–4 biological replicates. *p < 0.05, **p < 0.01, ***p < 0.001, ****p < 0.0001 vs. control; ^$^ *p* < 0.05, ^$$^ *p* < 0.01, ^$$$$^ *p* < 0.0001 vs. HG and ^#^ *p* < 0.05, ^####^ *p* < 0.0001 vs. CSE.

As shown in Figure 6a1, endothelial membrane expression of platelet endothelial cell adhesion molecule-1 (PECAM-1) was already up-regulated 12 h following exposure to HG and HG + CSE, as compared to control and CSE. Note the synergistic up-regulation of PECAM-1 expression following 12 h HG + CSE exposure when compared to HG and CSE conditions separately. The magnitude of the pro-inflammatory effect triggered by the tested conditions further increased at 24 h exposure where not only PECAM-1 overexpression became significantly higher than controls in all tested conditions (*p* < 0.05; see Figure 6b1), but also in HG + CSE vs. CSE and HG alone, thereby showing an additive effect. Besides PECAM-1, 12 h exposure to all the above mentioned experimental conditions triggered a statistically (*p* < 0.0001) significant up-regulation of intercellular adhesion molecule-1 (ICAM-1) in all the experimental conditions when compared to controls. In terms of magnitude, the effect was slightly diminished at 24 h exposure where only CSE and HG + CSE elicited a statistically significant effect on ICAM-1 expression (*p* < 0.05; see Figure 6a2, b2). By contrast to PECAM-1, ICAM-1 upregulation did not show an additive pattern.

ELISA analysis of the cell culture media following 24 h exposure to HG, CSE and HG + CSE revealed a significant upward trend (*p* < 0.05, *p* < 0.0001, and *p* < 0.0001 respectively) in the release of interleukin-8 (IL-8) compared to controls (Figure 6c2), which demonstrated an additive pattern when HG and CSE stimuli were combined. Interestingly, the release of interleukin-6 (IL-6) remained mostly unchanged except for a modest increase in HG + CSE culture conditions (Figure 6c1).

Nuclear-factor (erythroid derived 2) related factor-2 (Nrf2), one of the major transcription factors cooperatively regulating the anti-oxidant defense response was notably observed to be activated in endothelial cell cultures exposed to HG, CSE and HG + CSE. Further as shown by the Western blot analysis in Figure 6d there is a clear nuclear translocation of Nrf2 in response to all tested conditions. However, the effect was most significant in cultures exposed to either CSE or HG + CSE even when compared to HG alone. This is also evident in the graph bar depicting the ratios between nuclear vs. cytosol localization of this transcription factor.

## Discussion

Cerebrovascular pathological conditions such as stroke, Alzheimer’s, and multiple sclerosis involving a change in brain microenvironment are characteristically accompanied by BBB dysfunction [20], thereby emphasizing the necessity of maintaining a proper barrier function and integrity to conserve the brain tissue microenvironment. As mentioned earlier, both 2DM and TS have been considered to increase the chances of developing and progressing the above-mentioned cerebrovascular pathological conditions [1, 8–10]. Recent findings from our laboratory demonstrated that CSE exposure, comparable to physiological nicotine plasma concentrations of 100 ng/ml as observed in an average chronic smoker, induces BBB endothelial dysfunction and a strong inflammatory response. This included a down-regulation and redistribution of TJ protein expression, an up-regulation in expression of adhesion molecules and an increase in release of proinflammatory cytokines such as matrix metalloproteinase-2 (MMP-2) and IL-6 [25]. On similar lines, reports from our lab and others have demonstrated an altered expression and distribution of TJ and glucose transporter proteins in BBB endothelial cell cultures following exposure to DM like altered hyperglycemic conditions (35.0 mM d-glucose levels) [31].

As a TJ scaffolding protein anchored to the actin cytoskeleton, ZO-1 is crucial for the regulation of inter-endothelial TJ complexes and BBB structural integrity [42, 43]. Results from this study demonstrate a progressive down-regulation and disruption of ZO-1 expression/continuity at cell–cell contacts following 24 h exposure to HG, CSE or both, although an initial increase in expression of ZO-1 in membrane fractions was observed at 12 h under CSE conditions (Figure 2). These data further corroborate our previous studies indicating a 50% reduction in ZO-1 expression by exposure to CSE alone [25] and a marked disruption of ZO-1 bands at cell–cell contacts by HG [31]. In addition, diabetes-related hyperglycemia significantly suppressed ZO-1 expression in rodent BBB [44]. Based on these findings, it is likely that HG down-regulates ZO-1 expression with an apparent increase in its cytosolic redistribution [31]. However, we did not observe additive effects on ZO-1 reduction by HG + CSE exposure compared to either treatment alone, suggesting a saturated common pathway (Figure 2). Additionally, reduction of ZO-1 expression was accompanied by a decreased TEER across endothelial monolayers and significant increase in paracellular permeability to labeled dextrans in a size-selective manner, especially by CSE (Figure 3). This is in agreement with previous findings from our laboratory and others showing a proportionate increase in BBB permeability due to loss of TJ proteins [25, 31, 45]. Importantly, HG aggravated the CSE-induced BBB permeability of all labeled dextrans, thus implicating an exacerbated BBB damage by smoking co-morbid with diabetes. Moreover, as shown in Figure 3, loss of BBB integrity by CSE could also be plausibly explained by an increased endothelial release of VEGF, a potent mediator of BBB disruption that was previously shown to be involved in hyperglycemia–induced BBB disruption [31, 46]. Interestingly, our results correlate with previous findings [31] by Sajja et al. which have also shown only a modest increase of VEGF release from hCMEC/D3 monocultures whereas a significant higher output of this growth factor was observed in hCMEC/D3 co-cultures with human astrocytes. This is an additional experimental condition we are planning to exploit in future along with direct animal experimentation to validate our findings. Further, VEGF blockade restored the BBB integrity by preventing the loss of ZO-1 and other TJ proteins [47]. Thus, concomitant exposure to HG exacerbates CSE-induced BBB damage by potentiating VEGF release (Figure 3).

Glucose flux across the BBB is a dynamic phenomenon and is majorly mediated by facilitative and insulin-independent GLUT-1 (~55 kDa isoform) in response to the circulating glucose levels and cerebral metabolic demand [28]. In this study, we have shown for the first time that CSE exposure alone or concomitant with HG significantly increases the membrane expression of GLUT-1 in hCMEC/D3 cultures and the increase is sustained up to 24 h treatment (Figure 4). However, we observed a time-dependent effect (delayed onset) of HG on GLUT1 expression with an overall increase at 24 h but not 12 h exposure. Previously, conflicting lines of evidence were reported for the effects of hyperglycemia on BBB GLUT1 and glucose transport [28]. For example, experimental models of diabetes revealed a significant repression [48] or no change [49] in GLUT1 density and glucose transport at BBB. In this line, our recent findings have demonstrated unaltered total cellular GLUT1 (unfractionated) levels following 24 h exposure to hyperglycemic conditions in hCMEC/D3 cells [31]. In this study, we examined the changes in GLUT1 expression in membrane fractions isolated from cells exposed to HG and/or CSE. Given the increased membrane GLUT1 and unchanged total GLUT1 expression [31], we speculate that HG promotes the translocation and membrane deposition of cytosolic GLUT1. Additional functional studies are required to validate these findings. Nevertheless, our data shows a significant additive increase in membrane GLUT1 expression following combined exposure to HG and CSE, which would contribute to elevated levels of oxidative stress in BBB endothelium.

Additionally, our results indicate a significant and transient increase of SGLT-1 in hCMEC/D3 membranes following CSE alone or in combination with HG (Figure 5). As previously reported [31], prolonged exposure to HG (24 h) did not elicit significant changes in SGLT-1 expression. This is also in line with current findings showing that membrane expression of SGLT-1 return to baseline levels at 24 h following a transient increased at 12 h. Although the relative contribution of SGLT-1 to glucose transport at the BBB is currently unknown, it was previously shown that BBB SGLT-1 expression could be induced under pathophysiological conditions such as stroke [50] and hypoglycemia [31]. Therefore, it is likely that CSE-induced increase in SGLT-1 expression (12 h) in presence of HG could be mediated by an acute stressful response in hCMEC/D3 cells.

Diabetes-related hyperglycemia was previously shown to attenuate the expression of functional P-gp in rodent brain, thereby altering the CNS distribution of its substrates [51–53]. By contrast, other studies demonstrated a lack of effect [38] or a dramatic increase in P-gp expression [37] following hyperglycemia in mouse brain endothelial cells. Recently, we have shown that acute and chronic hyperglycemia differentially influences functional expression of BBB efflux transporters, including P-gp where repeated hyperglycemic stimuli over 3 days caused a significant increase in its efflux activity [54]. However, the effects of CSE alone and in the presence of HG on transporter activity of P-gp, remain unknown. Our data indicated a sustained up-regulation of membrane-bound P-gp expression by HG or CSE exposure following 24 h acute exposure although, no additive effects were observed under combined exposure conditions (Figure 5). Interestingly, P-gp activity (as measured by rhodamine123 efflux) was increased only by HG + CSE co-exposure at 24 h (as opposed to repetitive hits of HG exposure over several days as reported by Sajja et al.), suggesting a possible synergistic effect and an increased potential for altered CNS drug disposition of P-gp substrates in diabetic smokers [54]. It is also possible that repetitive stimuli [54] will further enhance this effect which we plan to test in future experiments. Given the extensive characterization of hCMEC/D3 cell line for its applicability to human BBB drug transport studies and its broad utility for understanding the molecular regulation of BBB efflux transporters in vivo [39, 55, 56], the findings of our study may hold clinical significance. However, in vivo validation studies will be necessary to confirm our results.

Existing evidence associates the role of inflammation and oxidative stress with the development of various CNS disorders in diabetes as well as chronic smoking. Previously we have shown that hyperglycemia induces transient up-regulation of inflammatory cell adhesion molecules such as VCAM-1 [31]. Similarly in a separate study we observed a moderate increase in the expression level of PECAM-1 and IL-8 following exposure to CSE from 3R4F cigarettes [25]. Herein we observed a significant increase of PECAM-1 in the co-exposure conditions suggesting an additive effect of HG and CSE. By contrast ICAM-1 was upregulated in all the conditions tested when compared to controls but no additive effect was observed. These results suggest the possibility for leukocyte-endothelial interactions eventually facilitating transmigration of circulating white blood cells (WBC) across the BBB [57]. This warrants further study in vivo to validate this hypothesis. In addition to vascular endothelial molecules, HG potentiated the release of IL-8 by CSE also strengthening the hypothesis for an additive inflammatory effect between HG and CSE prodromal to a possible neuroinflammatory disorder [58].

Nrf2 is master regulator and redox-sensitive transcription factor that has a pleiotropic role in mediating cellular antioxidant responses [59]. In fact we have recently shown the neuroprotective role of Nrf2 in regulating and maintaining BBB integrity [60]. As previously reported by our group hyperglycemia does not overall affect the total expression level of Nrf2 [31], however, HG modulates its distribution in the cells when we analyzed and compared the nuclear vs. cytosolic content. As shown in Figure 6d CSE exposure markedly elevated the nuclear/cytoplasmic ratio of Nrf2 suggesting activation of the antioxidant pathways. This effect was further enhanced by HG despite eliciting nuclear translocation of Nrf2 to a lesser extent when compared to CSE or HG + CSE. This latter implies the existence of a cooperative effect in term of cellular oxidative response activation. This in the long term can be more detrimental to BBB integrity if the oxidative stimuli overcome the protective antioxidant mechanisms of the BBB endothelial cells.

## Conclusions

Hyperglycemia exacerbates the pathological impact of cigarette smoke extract on BBB functional integrity in vitro, possibly through activation of endothelial inflammatory and oxidative stress responses. These hypotheses, however, need to be further validated in vivo to fully assess the pathophysiological effects in relation to the cerebrovascular system and onset of neuroinflammatory disorders.

## Abbreviations

ANOVA: analysis of variance
BBB: blood–brain barrier
CNS: central nervous system
EC: endothelial cell
ELISA: enzyme linked immunosorbent assay
EMB: endothelial basal medium
FBS: fetal bovine serum
FITC: fluorescein isothiocyanate
GLUT-1: glucose transporter-1
hCMEC/D3: immortalized human cerebromicrovascular endothelial cell/D3
HRP: horse radish peroxidase
ICAM-1: intercellular cell adhesion molecule-1
LDH: lactate dehydrogenase
IL-6: interleukin-6
Nrf-2: nuclear-factor (erythroid derived 2) related factor-2
PAGE: polyacrylamide gel electrophoresis
PBS: phosphate buffered saline
PECAM-1: platelet endothelial cell adhesion molecule-1
P-gp: P-glycoprotein
RITC: rhodamine B isothiocyanate
ROS: reactive oxygen species
SDS: sodium dodecyl sulfate
SGLT-1: sodium dependent glucose transporter-1
TBS: tris buffered saline
TEER: trans-endothelial electrical resistance
TJ: tight junction
VCAM-1: vascular cell adhesion molecule-1
VEGF: vascular endothelial growth factor
ZO-1: zonula occludens-1
